# Reduced Hornbill Abundance Associated with Low Seed Arrival and Altered Recruitment in a Hunted and Logged Tropical Forest

**DOI:** 10.1371/journal.pone.0120062

**Published:** 2015-03-17

**Authors:** Rohit Naniwadekar, Ushma Shukla, Kavita Isvaran, Aparajita Datta

**Affiliations:** 1 Nature Conservation Foundation, Mysore, Karnataka, India; 2 Centre for Ecological Sciences, Indian Institute of Science, Bangalore, Karnataka, India; Key Laboratory of Tropical Forest Ecology, Xishuangbanna Tropical Botanical Garden, Chinese Academy of Sciences, CHINA

## Abstract

Logging and hunting are two key direct threats to the survival of wildlife in the tropics, and also disrupt important ecosystem processes. We investigated the impacts of these two factors on the different stages of the seed dispersal cycle, including abundance of plants and their dispersers and dispersal of seeds and recruitment, in a tropical forest in north-east India. We focused on hornbills, which are important seed dispersers in these forests, and their food tree species. We compared abundances of hornbill food tree species in a site with high logging and hunting pressures (heavily disturbed) with a site that had no logging and relatively low levels of hunting (less disturbed) to understand logging impacts on hornbill food tree abundance. We compared hornbill abundances across these two sites. We, then, compared the scatter-dispersed seed arrival of five large-seeded tree species and the recruitment of four of those species. Abundances of hornbill food trees that are preferentially targeted by logging were two times higher in the less disturbed site as compared to the heavily disturbed site while that of hornbills was 22 times higher. The arrival of scatter-dispersed seeds was seven times higher in the less disturbed site. Abundances of recruits of two tree species were significantly higher in the less disturbed site. For another species, abundances of younger recruits were significantly lower while that of older recruits were higher in the heavily disturbed site. Our findings suggest that logging reduces food plant abundance for an important frugivore-seed disperser group, while hunting diminishes disperser abundances, with an associated reduction in seed arrival and altered recruitment of animal-dispersed tree species in the disturbed site. Based on our results, we present a conceptual model depicting the relationships and pathways between vertebrate-dispersed trees, their dispersers, and the impacts of hunting and logging on these pathways.

## Introduction

Hunting and logging are among the major threats affecting wildlife in tropical forests [1,2,3]. Many frugivorous animals that are affected by these threats play an important role in mutualistic relationships like seed dispersal. Seed dispersal plays an important role in the regeneration of plants and in governing tropical plant diversity. Hunting and logging often co-occur across many tropical forest sites. It is therefore important to understand the combined effects of these two threats on frugivores and on ecosystem processes like seed dispersal.

Hunting pressures on vertebrates in the tropics are often deemed unsustainable [4,5] and result in extreme low densities or extirpation of targeted species, such that the forests may remain structurally intact but become functionally defunct [6]. In tropical forests, seed dispersal is one of the key ecosystem processes that play an important role in governing plant diversity and regeneration. Frugivorous animals disperse seeds of up to 90% of tree species in tropical forests [7,8]. The loss of frugivores through hunting can affect seed dispersal in several ways—decreased frugivore visitation and lowered fruit removal [9,10], shorter dispersal distances, increased seed and/or seedling densities under parent plants resulting in density-dependent mortality. Thus loss of frugivores can affect the plant species composition resulting in lowered recruitment of biotically-dispersed plant species [11,12] and increased recruitment of abioticially dispersed species. Many large vertebrate frugivores targeted by hunting are important seed dispersers [2,13,14]. They are particularly important for large-seeded plants, which have fewer effective dispersers [15,16]. Logging, on the other hand, results in direct changes in forest structure [17], micro-climate [18] and reduced abundance of animal-dispersed plants [19,20]. While altered forest structure and micro-climate can have a bearing on plant recruitment, reduced abundance of animal-dispersed plants can result in reduced fruit availability of fruits thereby affecting frugivores indirectly. The lowered fruit availability could also lead to lower abundance and/or visitation by frugivores, which could lead to lower removal and seed dispersal for trees that continue to persist in logged landscapes.

Most of our current understanding of the impacts of hunting and logging on seed dispersal and plant recruitment is from the Neotropics [9,10,21,22,23], while there are very few studies from Asia [12,24,25]. Most studies have focused on studying one or few stages of the seed dispersal cycle [26] either during the fruit removal stage [9,10,22,27] or on comparisons of recruitment across hunted and control treatments [12,28,29,30]. Very few studies have looked at multiple stages in the seed dispersal cycle or tried to assess how anthropogenic disturbances could disrupt different stages in the cycle.

In this study we aimed to understand how logging and hunting, through the associated losses of fruiting trees and a key disperser group, affect scatter dispersal and recruitment patterns of large seeded forest trees. We focused on large-seeded tree species as they depend on large-bodied frugivores for dispersal. We selected hornbills, which are the largest avian frugivores and dispersers in Asian tropical forests [31] and five large-seeded tree species (*Phoebe* sp., *Canarium strictum*, *Beilschmiedia assamica*, *Dysoxylum* sp. and *Alseodaphne petiolaris*) as the model system for our study. These large-seeded tree species fruit during winter, the non-breeding season for hornbills [32]. Our study was carried out in the tropical forests of north-east India which face significant threats from logging and hunting [12,33,34]. Across most of this region, logging and hunting co-occur [12,33].

We expected that logging will negatively affect abundance of hornbill food plants, particularly those targeted by logging. This along with hunting, which results in direct removal of individuals from the population, will negatively affect the abundance of hornbills at the heavily disturbed site. We expected that reduced hornbill abundance would result in reduced arrival of scatter-dispersed seeds on forest floor. Given that sites have experienced these threats in the past, we expected to detect a signature of the negative impacts of hunting and logging on recruitment patterns—that is, reduced or altered recruit abundance in the heavily disturbed sites as compared to the less disturbed sites. Based on our results, we present a conceptual model depicting the relationships and feedbacks between vertebrate dispersed trees, their dispersers, and the two main threats to tropical biodiversity, hunting and logging.

## Materials and Methods

### Ethics statement

The office of the Principal Chief Conservator of Forests (Wildlife and Biodiversity), Government of Arunachal Pradesh, Itanagar approved the study and gave permission (No: CWL/G/13(17)06-07/Pt-III/4219-32) to conduct research in Namdapha Tiger Reserve (Protected Area) and Miao Reserved Forest in Arunachal Pradesh state.

### Study area

**Fig 1:**
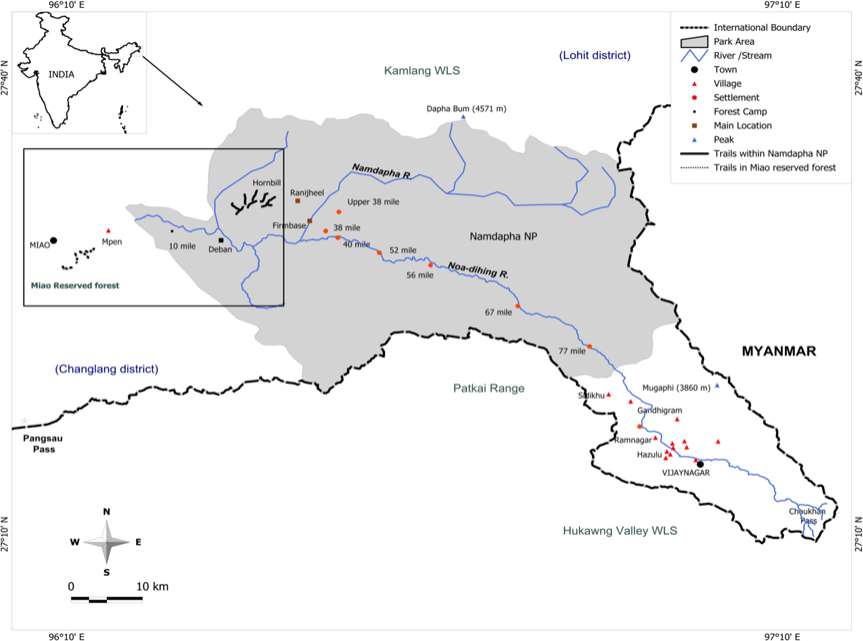
Map of study area. The area shaded gray is the Namdapha Tiger Reserve. The enclosed box shows the intensively sampled area with unbroken lines representing the eight trails in the less disturbed site (Namdapha Tiger Reserve) and broken lines representing the four trails in the heavily disturbed site (Miao Reserved Forest).

The study was conducted between November 2011 and March 2012 in the Namdapha Tiger Reserve (1985 km^2^; 200–4,500 m above sea level; 27°23’30”—27°39’40”N and 96°15’2”—96°58’33”E) and the adjoining Miao Reserved Forest (121 km^2^; 200–1300 m; 27°25’49”—27°30’02”N and 96°8’33”—96°18’59”E) in Arunachal Pradesh, north-east India (Fig. 1). The lower elevations of Namdapha harbor the world’s northernmost tropical wet evergreen rainforests [35,36]. The vegetation in the area is dominated by *Altingia excelsa*, *Terminalia myriocarpa*, *Dipterocarpus macrocarpus*, *Schima wallichii* and *Shorea assamica* [36].

More than 490 bird species have been recorded from Namdapha and adjoining areas [37]. Large avian frugivores with the ability to regurgitate large seeds are represented by five species of hornbills (Great Hornbill *Buceros bicornis*, Rufous-necked Hornbill *Aceros nipalensis*, Wreathed Hornbill *Rhyticeros undulatus*, White-throated Brown Hornbill *Anorrhinus austeni* and the Oriental Pied Hornbill *Anthracoceros albirostris*), Mountain Imperial Pigeon *Ducula badia*, Great Barbet *Megalaima virens*, Hill Myna *Gracula religiosa*, Green Cochoa *Cochoa viridis* and Purple Cochoa *Cochoa purpurea*. Some of the mammalian frugivores found in the area are Barking Deer *Muntiacus muntjak*, Sambar *Rusa unicolor*, Wild Pig *Sus scrofa*, Hoolock Gibbon *Hoolock hoolock*, Assamese Macaque *Macaca assamensis*, Stump-tailed Macaque *Macaca arctoides*, Northern Pig-tailed Macaque *Macaca leonina* and Rhesus Macaque *Macaca mulatta*.

Our study site, inside Namdapha Tiger Reserve (less disturbed site), was on a plateau (14 km^2^; 500–700 m above sea level) near the western border of the reserve. The other site in Miao Reserved Forest (heavily disturbed site) was c. 10 km^2^ with elevation ranging from 400 to 700 m and 20 km straight line distance from our study site in Namdapha.

In spite of a ban on hunting under the Wildlife (Protection) Act, 1972, [38] wildlife are primarily hunted for food, customary rituals and for trade in north-east India [34,39,40]. Hornbills are hunted for the upper beak and tail feathers by some tribes of the state of Arunachal Pradesh (Nyishi, Wancho, Tangsa and Nocte) to adorn their traditional headdresses. The two tribal communities (Lisu and Chakma) living around Namdapha do not specifically hunt hornbills for their body parts as they do not use them in traditional headdresses unlike other tribes. Namdapha was designated as a National Park and Tiger Reserve in 1983. Although its legal status as a Tiger Reserve acts as a deterrent to hunters, law enforcement inside the park is inadequate. Our study site was approximately 30 km from the nearest town (Miao) and eight km from the nearest village (Deban). Over a four-year period (2008–2012), we did not detect evidence of hornbill hunting inside the park (Rohit Naniwadekar, unpublished data). However, some hunting of other mammal groups like ungulates, primates and large carnivores is prevalent inside Namdapha. In India, timber harvest is banned inside Protected Areas and Namdapha has never been logged. Therefore, we classified Namdapha Tiger Reserve as a less disturbed site with no logging and low hunting pressures (mainly restricted to mammal species).

Miao Reserved Forest is located within one kilometer of Miao town, which is inhabited by several communities (Singpho, Tangsa, Wancho, Nyishi, Nocte, Adi, Lisu, Chakma and Nepali) with some of these tribes using hornbill body parts. Members of some of these tribes hunt ungulates, arboreal mammals, hornbills and small birds with muzzle loader guns (locally fabricated firearms), rifles, air guns or catapults (R. Naniwadekar, pers. obs.). In the past four years (2008–12), we recorded four instances of hunting of hornbills (three instances of Great Hornbills and one of Wreathed hornbills). Miao was notified as a Reserved Forest in 1962 and the earliest record of systematic logging operations is from 1978 (Working Plan, Jairampur Forest Division). A ban on logging through a Supreme Court order was in effect from 1996 till 2008 [41]. Logging started again in 2009. Selective logging for important timber species is allowed in designated forest areas [33,36], however, the harvest is often unsustainable [41] and has often led to complete loss of forested habitats [42]. For forestry operations, tree species are classified from Class A to F in decreasing order of value of timber. Two species of dipterocarps (*Dipterocarpus macrocarpus* and *Shorea assamica*) and *Terminalia myriocarpa* are the most valued timber species. Several hornbill food plants such as *Phoebe* spp., *Canarium strictum*, *Dysoxylum* sp., *Alseodaphne petiolaris*, *Aglaia* spp., *Cinnamomum glaucescens* and *Beilschmiedia* spp.[32] are also logged (S1 Table). We designated the Miao Reserved Forest as a heavily disturbed site because of logging, and hunting pressures on all vertebrate groups.

The two sites were similar to each other in geology, rainfall and elevation and forest/vegetation type. The straight-line distance between the furthest sampling sites was approximately 28 km (Fig. 1). We marked eight trails (each 1.5 km in length) and four trails each in the less disturbed and the heavily disturbed sites respectively. The trails were at least 500 m apart, except two trails in the less disturbed site that were 300 m apart (Fig. 1). However, we never walked two adjoining trails simultaneously. In the heavily disturbed site, we recorded all fallen logs or cut stumps GBH (girth at breast height) ≥ 100 cm within 20 m (10 m on each side) along each trail. We consistently recorded the number of hunters (individuals carrying guns) encountered while accessing, walking or returning from the trails for the two sites.

### Hornbill food plant abundance

We identified hornbill food plant species at both sites based on prior information [32]. We recorded all the known food plants including figs, non-fig trees with drupaceous or arillate dehiscent capsular fruits, which are consumed by hornbills in the non-breeding season (November—March) in 3 ha belt transects (1500 m × 20 m) along each trail in both sites. We classified the food plants into logged and not logged based on the list of timber species in the Working Plan of the Forest Department (S1 Table). We grouped strangler (hemi-epiphytic) figs that are not logged during selective logging operations separately. Hornbills also consume the fruits of the free-standing fig, *Ficus nervosa*, which is logged. We measured food plant abundance as the number of trees (GBH ≥ 30 cm) per 3 ha.

To model the effect of logging on hornbill food plants, we used Generalized Linear Models (GLM) on the count data of hornbill food plants with sites (less disturbed and heavily disturbed), type of hornbill food plant (logged food plants, not logged food plants and strangler figs), and an interaction between these two factors as predictor variables. We initially ran a GLM with Poisson errors but the results indicated over-dispersion in the data. Therefore, we used a negative binomial GLM with a log link.

### Hornbill encounter rates

We carried out trail walks at both sites between December 2011 and February 2012. In the less disturbed site, we walked trails in the mornings (0600–0900 hr) and late afternoons (1300–1500 hr), as hornbill activity is high during these time periods. We had eight trails, which were walked 12 times each. We largely avoided monitoring the same trail twice on the same day, except on seven occasions. In the heavily disturbed site, trails were walked only in the early mornings (0600–0900 hr) as human activity during the day could affect hornbill presence/detection in the late afternoons. One or two observers walked these trails and recorded hornbill species and number of individuals. The species identity was noted on hearing calls. However, we used only the visual detections for the analysis. We monitored each trail four times in a month. The total effort in less disturbed and heavily disturbed sites was 144 km and 72 km, respectively. Since we recorded only seven individuals in three detections in the heavily disturbed site in 72 km of effort, we were unable to estimate detection probability for the two sites separately. However, hornbills are large, striking and canopy-dwelling birds and are unlikely to be missed.

We used Generalized Linear Mixed-effect Models (GLMM) with Poisson errors to compare counts of hornbills sighted between less disturbed and heavily disturbed sites. There were differences in counts of hornbills across the three months (December = 173 individuals, January = 32 individuals, February = 41 individuals; effort = 48 km in each month in the less disturbed site), therefore, we incorporated ‘month’ effects as a random effect in the model. In addition, we walked each trail 12 times during the study duration, there was variation in the total number of hornbill sightings across the different trails (range: 2–105 individuals; effort: 18 km per trail). Therefore, we also used ‘trail’ as a random effect. In the heavily disturbed site, the total number of hornbill individuals seen was very low throughout the sampling period. The GLMM with trail and month effects as random and site effects (less disturbed and heavily disturbed) as fixed indicated over-dispersion in the data. We, therefore, incorporated individual observations as a random effect in the model following Elston et al. [43]. We used likelihood-ratio tests to test for the influence of random and fixed effects in GLMM.

### Seed arrival rates

In both sites along each trail, we established 200 (1 m × 1m) plots on the forest floor to record the arrival of scatter-dispersed seeds (1600 m^2^ in less disturbed site; 800 m^2^ in heavily disturbed site). We monitored these plots every 15 days (except once when the monitoring interval was 29–31 days) from December 2011–February 2012 (five occasions). We monitored the arrival of dispersed seeds of five large-seeded hornbill food plants—*Canarium strictum*, *Phoebe* sp., *Beilschmiedia assamica*, *Alseodaphne petiolaris* and *Dysoxylum* sp. in these plots (Table 1). After enumeration, we removed all the accumulated seeds. We considered the seeds without any trace of pulp as having been dispersed. We recorded the species identity and the number of seeds in the plot. We did not use above-ground netted seed traps because during an earlier study in the area, our nets had been taken away by people or damaged. While on-ground seed removal/predation by rodents may potentially occur, affecting the seed arrival estimates, this is unlikely to affect our study results. First, we had set out paired 1 m^2^ above-ground netted seed traps and ground plots (100 each) and monitored seed arrival rates over 42 days in the same season in 2010–11. Seed arrival rates were estimated to be 0.001 per m^2^ per day for seed traps and 0.002 per m^2^ per day for ground plots for three of the five study species (*C*. *strictum*, *B*. *assamica* and *Dysoxylum* sp.). Hornbills were amongst the most common frugivores in terms of frequency of visits and the number of individuals that visited these five species. Additionally, as compared to smaller frugivores like barbets and cochoas, they swallowed a greater proportion of fruits they handled [44]. Second, in a pilot study to estimate rodent seed removal rates, we had set out 400 marked seeds (five seeds each in ten 1 m^2^ plots for eight trails) and found that average seed removal was relatively low (14.8%) for one of our study species, *C*. *strictum*. We do not have information on rodent abundances at the two sites, however, some of the local people hunt rodents in the heavily disturbed site both with firearms and traps, while there are no hunting pressures on rodents in the less disturbed site, therefore it is unlikely that there would have been greater seed removal by rodents at the heavily disturbed site which could affect seed arrival estimates there.

**Table 1:** Mean (SE) of the fruit and seed length (mm) and width (mm) for the five large-seeded hornbill food plants.

Tree species	Fruit length	Fruit width	Seed length	Seed width
*Phoebe* sp.	36.5 (0.9)	28.8 (1.3)	27.8 (3.6)	16.9 (0.96)
*Beilschmiedia assamica*	36.7 (0.4)	27.4 (0.1)	34.3 (2.8)	22.9 (1.2)
*Dysoxylum* sp.	30.4 (0.5)	18.3 (0.6)	28.3 (0.95)	17.2 (0.5)
*Canarium strictum*	40.6 (0.8)	24.7 (0.6)	33.8 (0.96)	15.1 (1.1)
*Alseodaphne petiolaris*	43.3 (0.7)	21.8 (0.4)	35.6 (2.2)	17.4 (1.0)

Although seed arrival rates at the less disturbed site were similar between the two years, they differed in species composition [44]. These differences are likely to be due to supra-annual variation in fruiting of these species [45]. Therefore, we did not compare seed arrival rates of individual species at less disturbed and heavily disturbed sites, but compared the overall arrival rates of large seeds of the five focal tree species between both sites. We used GLMM with Poisson errors to compare seed arrival between less disturbed and heavily disturbed sites. There was considerable variation in total number of seeds detected in plots across trails (range: 2–107) and the different monitoring sessions (range: 4–92). We incorporated trails and the different monitoring sessions as random effects. We used natural logarithms of time interval (number of days) between monitoring sessions as offsets to control for differences in time interval between monitoring sessions and trails. The model indicated over-dispersed data. We therefore used individual observations as a random effect.

### Recruitment of hornbill food plants

Along each trail at both sites, we established belt transects measuring 1500 m × 3 m. For two trails in the less disturbed site, we sampled 750 m × 3 m. We recorded recruits of four of the five large-seeded hornbill food plants, *Canarium strictum*, *Phoebe* sp., *Beilschmiedia assamica* and *Dysoxylum* sp. We were not able to identify recruits of *Alseodaphne petiolaris*. We recorded the species identity and number of individuals of the recruits and measured the size to compare the size structure (seedling: 10–30 cm, large seedling: 30–50 cm, sapling: 50–100 cm, large sapling: 100–150) of the recruits between the two sites.

To compare the recruits in four size classes between the less disturbed and the heavily disturbed site, we used GLM on the count data of recruits with sites (less disturbed and heavily disturbed), size of the recruits (10–30 cm, 30–50 cm, 50–100 cm, 100–150 cm) and an interaction between these two factors as predictor variables. We used the natural logarithm of area sampled as an offset to control for varying sampling effort between trails. We detected only 6 and 10 recruits of *Canarium strictum* across all size classes at less disturbed and heavily disturbed sites respectively; therefore, we did not perform statistical analysis for this species. We initially ran a GLM with Poisson errors but the results indicated over-dispersion in the data, therefore, we used a negative binomial GLM with a log link.

We carried out all the analysis using R Language, version 2.15.1 [46]. We used the package ‘MASS’ for carrying out the negative binomial GLMs and package ‘lme4’ [47] for the GLMMs.

## Results

### Logging and hunting pressures

In the heavily disturbed site, the density of cut logs/stumps (GBH≥100 cm) was 11±1 logs/stumps per ha (mean ± SD). There was no logging in the less disturbed site. Despite a lower sampling effort in the heavily disturbed site, we recorded hunters (men with guns) on six occasions, while we heard human presence only once in the less disturbed site. Additionally, we saw feathers of Wreathed Hornbills on the forest floor next to a temporary camp in the heavily disturbed site, outside of the trail sampling.

### Hornbill food plant abundance

We identified six species of figs and 15 species of non-fig food plants (S1 Table). Of the 21 hornbill food plant species, ten were timber species (S1 Table).

**Fig 2:**
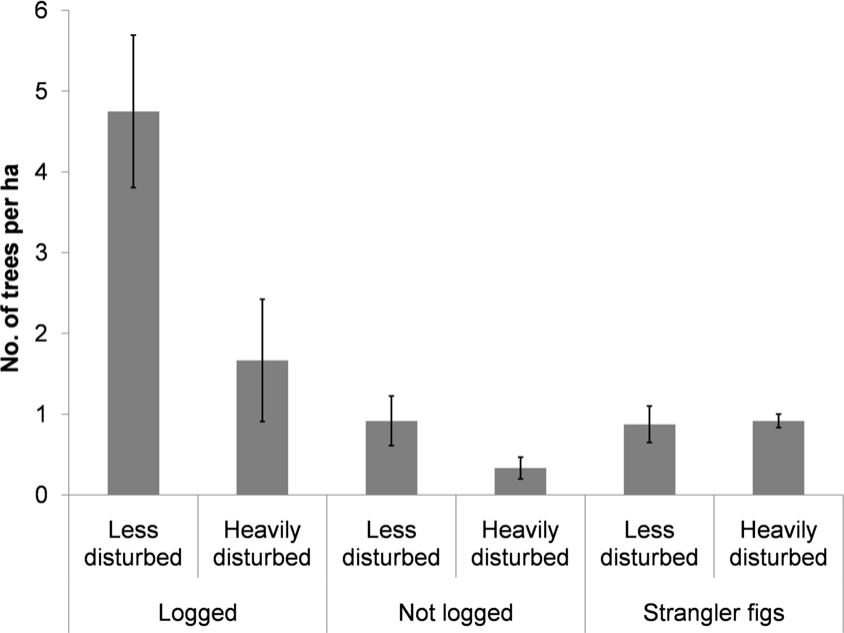
Number of trees per ha (± SE) across the three different categories of food plants. (a) logged, food plants (b) not logged and (c) strangler figs in the less disturbed site (Namdapha) and the heavily disturbed site (Miao).

The overall abundance of hornbill food plants was two times higher in the less disturbed site as compared to the heavily disturbed site (negative binomial GLM, *z*_1, 10_ = -2.562, *p* = 0.01; Fig. 2). The abundance of food plants that were also timber species was significantly higher than non-timber food plants (*z*_1, 10_ = -5.373, *p*<0.001; Fig. 2; S2 Table) and strangler figs (*z*_1, 10_ = -4.757, *p* = 0.01; Fig. 2; S2 Table) in both sites. Two-way interactions between site and type of food plant were not significant (negative binomial GLM, *p*>0.05). The mean densities of the five large-seeded species that are also the important hornbill food plants was higher in the less disturbed site as compared to the heavily disturbed site (Table 2).

**Table 2:** Mean (± SE) of adult tree densities (per ha) and seed arrival rates (per ha) on forest floor of five important hornbill food plant species.

Tree species	Tree density in less disturbed	Tree density in heavily disturbed	Seed arrival in less disturbed	Seed arrival in heavily disturbed
*Phoebe* sp.	0.375 (0.292)	0.167 (0.167)	863 (525)	0 (0)
*Beilschmiedia assamica*	2.458 (0.804)	0.250 (0.160)	256 (71)	0 (0)
*Dysoxylum* sp.	1.500 (0.508)	0.000 (0.000)	50 (25)	0 (0)
*Canarium strictum*	0.042 (0.042)	0.25 (0.25)	38 (16)	13 (13)
*Alseodaphne petiolaris*	0.125 (0.061)	0.083 (0.083)	306 (92)	150 (74)

### Hornbill encounter rates

**Fig 3:**
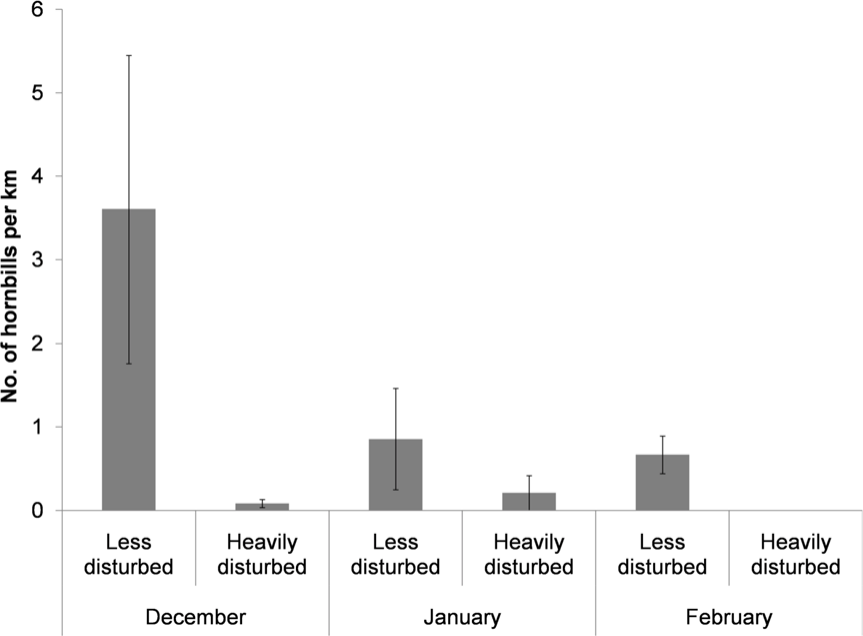
Number of hornbills per km (± SE) detected across the sampling period in the less disturbed site (Namdapha) and the heavily disturbed site (Miao).

During trail walks, we detected four species of hornbills (Great, Wreathed, Rufous-necked and Brown Hornbill) in the less disturbed site and two species (Wreathed and Rufous-necked Hornbill) in the heavily disturbed site. The total number of hornbills seen across the trails varied from 2–105 individuals at the less disturbed site and 0–6 individuals at the heavily disturbed site. The total number of individuals detected varied across months in the less disturbed site (December: 173, January: 41 and February 32). Overall encounter rates of hornbills was 22 times higher in the less disturbed site as compared to the heavily disturbed site (*z*_1, 10_ = -3.123, *p* = 0.002; Fig. 3; S3 Table).

### Seed arrival of food plant species

**Fig 4:**
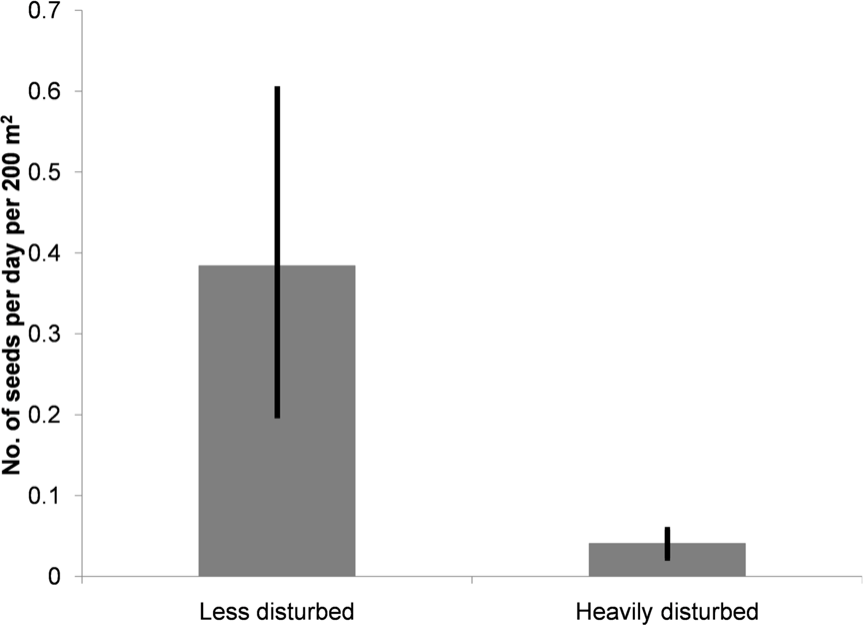
Seed arrival per day in heavily and less disturbed sites. Bootstrap mean and 95% confidence intervals of number of seeds per day per 200 m^2^ (200 1 m^2^ plots per trail) in the less disturbed site (Namdapha) and the heavily disturbed site (Miao).

We detected seeds of all the five species (*Phoebe* sp., *Canarium strictum*, *Beilschmiedia assamica*, *Alseodaphne petiolaris*, and *Dysoxylum* sp.) at the less disturbed site and only two species (*Canarium strictum* and *Alseodaphne petiolaris*) at the heavily disturbed site. The mean arrival rate of the each of the five species was higher in the less disturbed site as compared to the heavily disturbed site (Table 2). Scatter-dispersed seed arrival was seven times higher at the less disturbed site as compared to heavily disturbed site (*z*_1, 10_ = -2.366, *p* = 0.018; Fig. 4; S4 Table).

### Recruitment of food plant species

**Fig 5:**
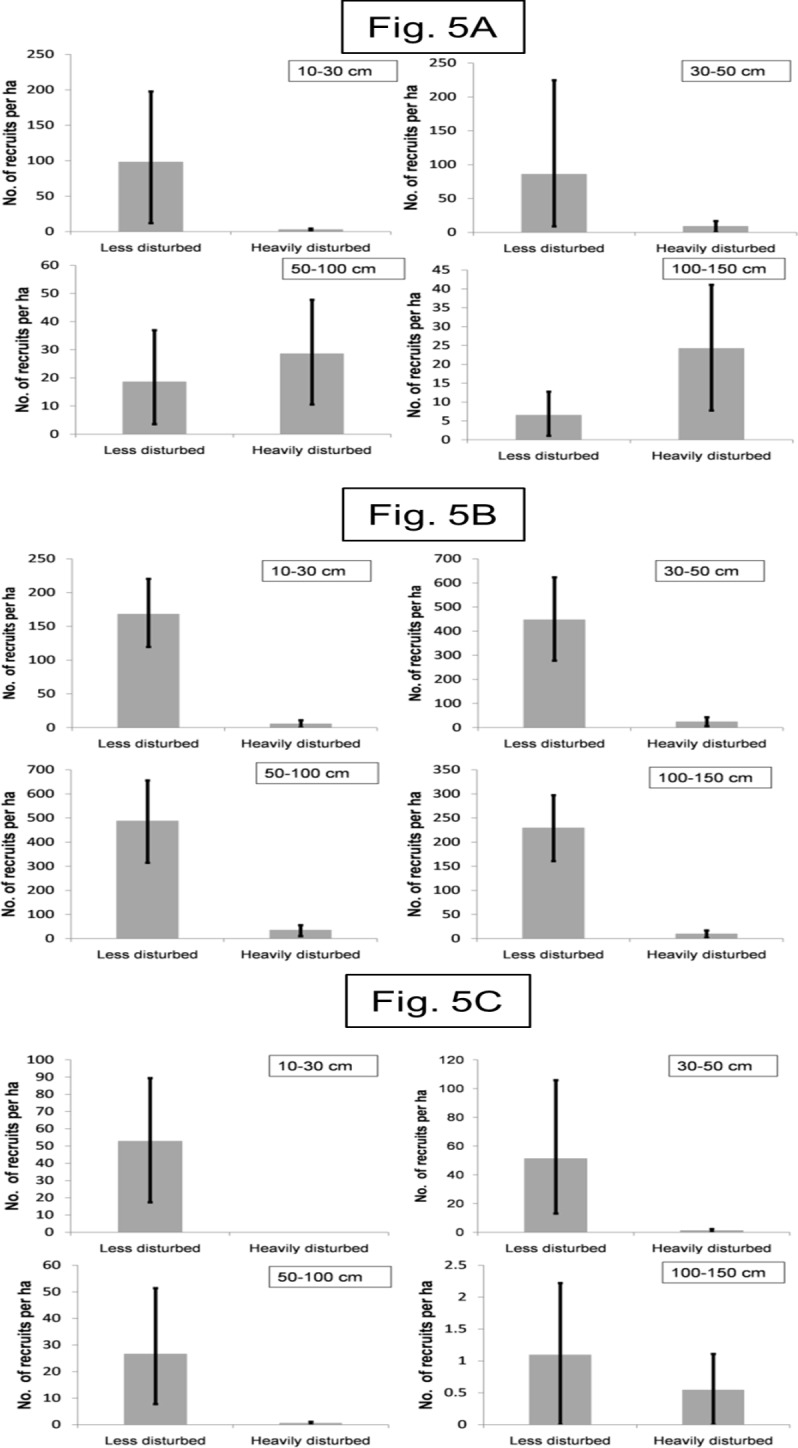
Seedling and sapling recruitment in heavily and less disturbed sites. Bootstrap mean and 95% confidence intervals of number of recruits per ha in the less disturbed site (Namdapha) and the heavily disturbed site (Miao) for a) *Phoebe* sp. b) *Beilschmiedia assamica* and c) *Dysoxylum* sp. across the four size classes 10–30 cm, 30–50 cm, 50–100 cm and 100–150 cm.

The overall abundance of recruits across all size classes was 20 times higher for *B*. *assamica* and 48 times higher for *Dysoxylum* sp. in the less disturbed site as compared to the heavily disturbed site (Fig. 5B and Fig. 5C; S5 Table). The interaction term between size and abundance was not significant in these two species (Poisson GLM, two-way interaction between site and size of recruits was not significant *p*> 0.05). The difference in abundance between less disturbed and heavily disturbed sites was similar for recruits of all size classes in these two species. In contrast, for *Phoebe* sp., the difference between the abundance of small and large-sized recruits was significantly higher in the heavily disturbed site as compared to less disturbed site (Fig. 5A; S5 Table).

## Discussion

**Fig 6:**
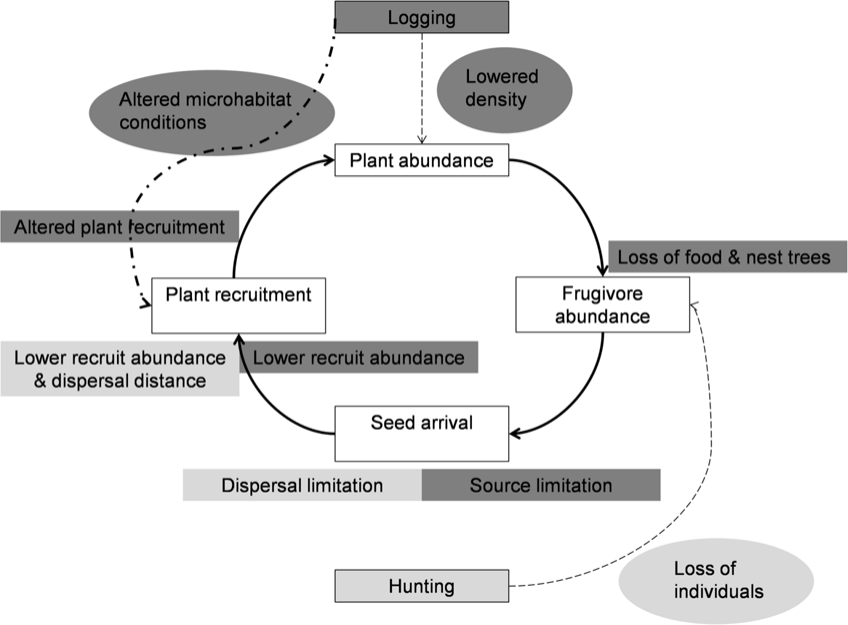
Conceptual model of direct and indirect impacts of logging and hunting on seed dispersal cycle. The relationship (black solid arrow) across different stages of seed dispersal (shown in open rectangular boxes) and direct (color coded oval box without outline and broken arrow) and indirect (color coded rectangular box without outline) impacts of logging (dark grey rectangular box with outline) and hunting (light grey rectangular box with outline) on the different stages of the seed dispersal. Dashed and dotted line shows additional likely impacts of logging (not explored in this study) on plant recruitment.

We found that the heavily disturbed site had reduced abundances of fruit plants, hornbills and scatter-dispersed seeds, and showed altered recruitment patterns. These findings suggest that logging can result in lowered abundance of hornbill food tree species indirectly affecting hornbill abundance, scatter-dispersal of seeds and their recruitment. Hunting can result in direct reduction of dispersers like hornbills, indirectly affecting scatter-dispersal of seeds and their recruitment. Our inferences are based on comparing two similar tropical forest sites close to each other (~ 20 km) that primarily differed in the extent of hunting and logging. Due to the absence of multiple comparable sites representing the less disturbed scenario, we did not have more replicate sites. However, we found that several different stages of the seed dispersal cycle consistently showed differences between our two sites as expected by the hypothesized effects of hunting and logging. This suggests that our findings were robust and that logging and hunting underlie these differences. Based on the findings of this study and other expected impacts, we propose a conceptual model that outlines the direct and indirect impacts of logging and hunting on the seed dispersal cycle (Fig. 6).

In this study, we compared four different stages of the seed dispersal cycle—abundance of food plants, abundance of frugivores, net seed arrival and recruitment patterns of selected large-seeded biotically dispersed plants (Fig. 6, open rectangular boxes) across heavily disturbed and less disturbed sites. Logging and hunting, are expected to differentially impact these four stages. Logging is expected to have direct impacts (Fig. 6, oval box) on the abundances of plants and indirect impacts on frugivores through reduced resource availability (Fig. 6, dark grey box). While the frugivore guild is known to be vulnerable to anthropogenic perturbations including logging [48,49,50], studies that have documented reduction in resource availability as a consequence of these impacts are few (however see [19,20]). In our study site, timber species that are targeted by logging include both abiotically-dispersed species (for e.g. *Terminalia myriocarpa*, *Dipterocarpus macrocarpus*, *Shorea assamica*) and biotically-dispersed species (including some species in this study). Logging of biotically-dispersed species can reduce the abundance of food plants as has been documented in this study. In our study site, abundance of hornbill food plant species that are logged is significantly higher than the abundance of food plants that are not logged highlighting the considerable reduction in fruit availability as a consequence of logging. This is despite the species richness of plants across the three categories being similar (strangler figs: 5 spp., not logged: 6 spp., logged: 9 spp.). Amongst the hornbill food plants that are targeted for logging, *Canarium strictum* and *Phoebe* sp. are two very important hornbill food plant species. Unlike many other non-fig food plants, they are emergent trees with large fruit crop sizes and have a high representation in hornbill diet. In 2010–11, *Canarium strictum* was represented in 50% and 19% of the foraging sightings of the Wreathed and Rufous-necked Hornbills [44]. In 2011–12, when *Canarium strictum* did not fruit, *Phoebe* sp. was among the most important hornbill food plants [44]. Additionally, our data from Namdapha, the less disturbed site, indicates that areas with higher abundance of these two species attract higher abundances of hornbills and have higher diversity of recruits of large-seeded hornbill food plants. Loss of such plants to logging can therefore be expected to have detrimental effects on hornbills and recruitment patterns of other fruiting plants. While abiotically-dispersed species that are targeted by logging at our site are not hornbill food plants, some of them belonging to genus *Terminalia*, *Dipterocarpus*, and *Shorea* are important hornbill nesting trees. Logging of these species can negatively affect the availability of nesting sites. These hardwood genera are known to be used by hornbills for nesting in Namdapha and other areas in south-east Asia [16,51]. This aspect was not examined in the study but is outlined in the conceptual model (Fig. 6).

In the short-term, hunting will have no impacts on food plant abundance. Hunting of frugivores will result in their reduced abundances in the ecosystem (Fig. 6). Amongst the different avian frugivores, hornbills are the largest, they occur in lower densities, have slow breeding rates and take as many as four years to reach sexual maturity [16,52]. Large-bodied hornbills usually raise a single chick in a year [52]. This makes hornbills vulnerable to hunting pressures exerted for their meat and body parts (tail feathers and casque) which are used by local communities in north-east India and south-east Asia for traditional reasons [39,53]. In this study, we found a 22-fold decrease in hornbill abundance in the heavily disturbed site as compared to the less disturbed site. A combination of both logging and hunting can be expected to result in lower abundances of hornbills in the heavily disturbed site. While logging would have indirect impacts on hornbills through reduced fruit availability, hunting results in direct negative impacts on hornbill populations in the area. Given the difficulty in finding sites with only hunting or only logging in eastern Arunachal Pradesh, we are unable to differentiate between the relative impacts of hunting and logging on hornbills.

Given that logging likely affects the abundance of food plants of frugivores and hunting impacts the frugivores themselves, negative impacts on the further stages of seed dispersal cycle of frugivore-dispersed trees, including seed dispersal and recruitment, can be expected. Scatter dispersal of seeds as has been documented in this study is mediated by frugivores through fruit removal. While studies have documented reduced frugivore visitation, especially large-bodied frugivores in sites experiencing anthropogenic disturbances [9,22,24], estimates of seed arrival of scatter-dispersed seeds have been rarely quantified. In Namdapha, we consistently found positive relationships between hornbill abundance and net seed arrival of four large-seeded plants across two years [44]. Thus loss of hornbills is expected to reduce net scatter dispersal of seeds on the forest floor. Supporting this expectation, we found 7-fold higher seed arrival in the less disturbed site as compared to the heavily disturbed site. The observed reduced net arrival of scatter-dispersed seeds could be a consequence of both dispersal and source-limitation. Logging caused reduction in abundance of adult trees, which would result in lowered fruit availability for scatter-dispersal indirectly affecting scatter-dispersal of seeds. Logging and hunting affect hornbill abundance, which will directly result in reduced scatter-dispersal of seeds (Fig. 6). In our study, we measured net arrival of seeds (as the arrival of seeds was recorded in plots and not in seed traps), we have not measured the actual seed rain but have measured seeds that remain on the forest floor (net arrival of scatter-dispersed seeds). Secondary dispersal by rodents can be expected to influence this net arrival of scatter-dispersed seeds differentially across the less and heavily disturbed sites. However, rodents and squirrels face hunting pressures in the heavily disturbed site but not in the less disturbed site. Loss of rodents to hunting is known to reduce secondary seed dispersal and seed predation by rodents. In such a scenario, differences in estimates of net seed arrival in heavily and less disturbed site, are likely to be conservative. Forests experiencing anthropogenic threats like hunting and logging demonstrate shifts in recruit communities with greater representation of abiotically dispersed species as compared to biotically dispersed species [24,29]. We found that disturbed forests experience lowered recruitment of two large-seeded species *Beilschmiedia assamica* and *Dysoxylum* sp. This has also been found in other studies in several tropical forest sites [12,21,28]. Large-seeded biotically dispersed species are known to be particularly vulnerable to these threats as they have a smaller assemblage of frugivores as compared to small-seeded species [24,28,48]. Interestingly, for *Phoebe* sp., we found a significant interaction between disturbance and recruit size. In the lower size classes, the abundance of recruits was higher in the less disturbed site while in the higher size classes the pattern was reversed. This could be a consequence of poor recruitment in the recent past due to source and dispersal limitation in the heavily disturbed site. For *Canarium strictum*, the abundance of recruits was extremely low as compared to other species at both the heavily and less disturbed site. Recruitment of plants is also dependent on a suite of other factors like micro-habitat conditions [54,55] and environmental factors [56]. In fact, improved light conditions are known to enhance recruitment in most (75%) of the plants at a tropical forest site in Panama [55]. Thus while logging can reduce recruitment patterns via source limitation on one hand, it can also enhance recruitment of plants through improved light conditions on the forest floor (Fig. 6). Therefore in human-modified landscapes, resilience of species to different anthropogenic disturbances can be expected to vary as seen for the four tree species at our study site.

Reduced recruitment in the disturbed site of certain high value timber species that are animal-dispersed has consequences for long-term persistence of these species and affect the sustainability of timber harvests in logged forests in the long-term. Given that these logged forests also experience hunting of important seed dispersers like hornbills, the negative impacts on the timber species dependent on large vertebrates for seed dispersal are exacerbated. Given that even the less destructive practices like reduced-impact logging [3,17,24,57], are detrimental to large frugivores [58], it is therefore urgently necessary to re-evaluate and modify current logging practices in the tropical forests of north-east India that ensure the persistence of these intricate relationships between plants and animals.

## Supporting Information

S1 Table List of hornbill food plants and their timber class. Hornbill food plants were categorized into strangler figs, hornbill food plants, which are logged, and hornbill food plants that are not logged. Class A-E (as per the Working Plan, Jairampur Forest Division) represents decreasing order of preference for timber value.
(DOCX)

S2 Table Abundance of hornbill food plants across disturbance types. Results from the GLM with negative binomial errors comparing hornbill food plant abundance across three categories (food plants which are logged, food plants which are not logged and strangler figs) between the two sites (Namdapha—with no logging and low hunting pressures and Miao—with logging and high hunting pressures). Parameter estimates (intercept and contrasts), standard errors (SE) and hypothesis tests for parameters are shown.
(DOCX)

S3 Table Hornbill abundance across disturbance types. Results from the GLMM with Poisson errors comparing hornbill abundance between Namdapha (with no logging and low hunting pressures) and Miao (with logging and high hunting pressures). Parameter estimates (intercept and contrast), standard errors (SE) and hypothesis tests for parameters are shown.
(DOCX)

S4 Table Scatter-dispersed seed abundance across disturbance types. Results from the GLMM with Poisson errors comparing abundance of scatter-dispersed seeds between Namdapha (with no logging and low hunting pressures) and Miao (with logging and high hunting pressures). Parameter estimates (intercept and contrast), standard errors (SE) and hypothesis tests for parameters are shown.
(DOCX)

S5 Table Recruit abundance across disturbance types. Results from the GLM with negative binomial errors, for *Beilschmiedia assamica*, *Phoebe* sp. and *Dysoxylum* sp., comparing recruit abundance between Namdapha (with no logging and low hunting pressures) and Miao (with logging and high hunting pressures) and four size classes (10–30 cm, 30–50 cm, 50–100 cm and 100–150 cm). Three orthogonal contrasts were set for comparisons. Contrast 1: size 10–30 cm vs. other size classes (30–50 cm, 50–100 cm and 100–150 cm), contrast 2: size 30–50 cm vs. other size classes (50–100 cm, 100–150 cm) and contrast 3: size 50–100 cm vs. size 100–150 cm. Parameter estimates (intercept and contrast), standard errors (SE) and hypothesis tests for parameters are shown.
(DOCX)
