# Supplementary material for: Reduced Hornbill Abundance Associated with Low Seed Arrival and Altered Recruitment in a Hunted and Logged Tropical Forest
Source: PLoS One. 2015 Mar 17;10(3):e0120062. doi: 10.1371/journal.pone.0120062 (PMC4363152; doi:10.1371/journal.pone.0120062)
Supplement: S1 Table — Hornbill food plants were categorized into strangler figs, hornbill food plants, which are logged, and hornbill food plants that are not logged. Class A-E (as per the Working Plan, Jairampur Forest Division) represents decreasing order of preference for timber value. (DOCX) [file pone.0120062.s001.docx]

**S1 Table.** **List of hornbill food plants and their timber class.** Hornbill food plants were categorized into strangler figs, hornbill food plants, which are logged, and hornbill food plants that are not logged. Class A-E (as per the Working Plan, Jairampur Forest Division) represents decreasing order of preference for timber value.

| Category | Family | Tree species | Timber Class |
| --- | --- | --- | --- |
| Logged | Lauraceae | *Phoebe* sp. | A/B |
| Logged | Burseraceae | *Canarium strictum* | B |
| Logged | Lauraceae | *Phoebe cooperiana* | B |
| Logged | Meliaceae | *Dysoxylum* sp. | B |
| Logged | Meliaceae | *Aglaia spectabilis* | B |
| Logged | Lauraceae | *Alseodaphne petiolaris* | C |
| Logged | Meliaceae | *Aglaia* sp. | C |
| Logged | Lauraceae | *Beilschmiedia assamica* | E |
| Logged | Lauraceae | *Beilschmiedia* sp. | E |
| Logged | Moraceae | *Ficus nervosa* | E |
| Strangler fig | Moraceae | *Ficus microcarpa* | Non-timber |
| Strangler fig | Moraceae | *Ficus drupacea* | Non-timber |
| Strangler fig | Moraceae | *Ficus altissmia* | Non-timber |
| Strangler fig | Moraceae | *Ficus* cf. *tsjahela* | Non-timber |
| Strangler fig | Moraceae | *Ficus* sp. | Non-timber |
| Not logged | Annonaceae | *Polyalthia* cf. *simiarum* | Non-timber |
| Not logged | Lauraceae | *Machilus duthiei* | Non-timber |
| Not logged | Lauraceae | *Cryptocarya* sp. | Non-timber |
| Not logged | Lauraceae | *Actinodaphne angustifolia* | Non-timber |
| Not logged | Oleaceae | *Chionanthus* sp.? | Non-timber |
| Not logged | Rosaceae | *Prunus ceylanica* | Non-timber |
