# Supplementary material for: Reduced Hornbill Abundance Associated with Low Seed Arrival and Altered Recruitment in a Hunted and Logged Tropical Forest
Source: PLoS One. 2015 Mar 17;10(3):e0120062. doi: 10.1371/journal.pone.0120062 (PMC4363152; doi:10.1371/journal.pone.0120062)
Supplement: S2 Table — Results from the GLM with negative binomial errors comparing hornbill food plant abundance across three categories (food plants which are logged, food plants which are not logged and strangler figs) between the two sites (Namdapha—with no logging and low hunting pressures and Miao—with logging and high hunting pressures). Parameter estimates (intercept and contrasts), standard errors (SE) and hypothesis tests for parameters are shown. (DOCX) [file pone.0120062.s002.docx]

**S2 Table.** **Abundance of hornbill food plants across disturbance types.** Results from the GLM with negative binomial errors comparing hornbill food plant abundance across three categories (food plants which are logged, food plants which are not logged and strangler figs) between the two sites (Namdapha – with no logging and low hunting pressures and Miao – with logging and high hunting pressures). Parameter estimates (intercept and contrasts), standard errors (SE) and hypothesis tests for parameters are shown.

|  | Estimate | SE | *z* | *P* |
| --- | --- | --- | --- | --- |
| Intercept (Site – Namdapha; Category – Logged) | 2.5683 | 0.1891 | 13.583 | <0.001 |
| Site – Miao | -0.6996 | 0.2731 | -2.562 | 0.01 |
| Category – Not logged | -1.6222 | 0.3019 | -5.373 | <0.001 |
| Category – Strangler fig | -1.3635 | 0.2866 | -4.757 | <0.001 |
