# Supplementary material for: Reduced Hornbill Abundance Associated with Low Seed Arrival and Altered Recruitment in a Hunted and Logged Tropical Forest
Source: PLoS One. 2015 Mar 17;10(3):e0120062. doi: 10.1371/journal.pone.0120062 (PMC4363152; doi:10.1371/journal.pone.0120062)
Supplement: S4 Table — Results from the GLMM with Poisson errors comparing abundance of scatter-dispersed seeds between Namdapha (with no logging and low hunting pressures) and Miao (with logging and high hunting pressures). Parameter estimates (intercept and contrast), standard errors (SE) and hypothesis tests for parameters are shown. (DOCX) [file pone.0120062.s004.docx]

**S4 Table.** S**catter-dispersed seed abundance across disturbance types.** Results from the GLMM with Poisson errors comparing abundance of scatter-dispersed seeds between Namdapha (with no logging and low hunting pressures) and Miao (with logging and high hunting pressures). Parameter estimates (intercept and contrast), standard errors (SE) and hypothesis tests for parameters are shown.

|  | Estimate | SE | *z* | *P* |
| --- | --- | --- | --- | --- |
| Intercept (Site – Namdapha) | -2.0489 | 0.5363 | -3.82 | <0.001 |
| Site – Miao | -1.9405 | 0.8201 | -2.366 | 0.018 |
