# Supplementary material for: Reduced Hornbill Abundance Associated with Low Seed Arrival and Altered Recruitment in a Hunted and Logged Tropical Forest
Source: PLoS One. 2015 Mar 17;10(3):e0120062. doi: 10.1371/journal.pone.0120062 (PMC4363152; doi:10.1371/journal.pone.0120062)
Supplement: S5 Table — Results from the GLM with negative binomial errors, for Beilschmiedia assamica, Phoebe sp. and Dysoxylum sp., comparing recruit abundance between Namdapha (with no logging and low hunting pressures) and Miao (with logging and high hunting pressures) and four size classes (10–30 cm, 30–50 cm, 50–100 cm and 100–150 cm). Three orthogonal contrasts were set for comparisons. Contrast 1: size 10–30 cm vs. other size classes (30–50 cm, 50–100 cm and 100–150 cm), contrast 2: size 30–50 cm vs. other size classes (50–100 cm, 100–150 cm) and contrast 3: size 50–100 cm vs. size 100–150 cm. Parameter estimates (intercept and contrast), standard errors (SE) and hypothesis tests for parameters are shown. (DOCX) [file pone.0120062.s005.docx]

**S5 Table.** **Recruit abundance across disturbance types.** Results from the GLM with negative binomial errors, for *Beilschmiedia assamica*, *Phoebe* sp. and *Dysoxylum* sp., comparing recruit abundance between Namdapha (with no logging and low hunting pressures) and Miao (with logging and high hunting pressures) and four size classes (10-30 cm, 30-50 cm, 50-100 cm and 100-150 cm). Three orthogonal contrasts were set for comparisons. Contrast 1: size 10-30 cm vs. other size classes (30-50 cm, 50-100 cm and 100-150 cm), contrast 2: size 30-50 cm vs. other size classes (50-100 cm, 100-150 cm) and contrast 3: size 50-100 cm vs. size 100-150 cm. Parameter estimates (intercept and contrast), standard errors (SE) and hypothesis tests for parameters are shown.

| *Beilschmiedia assamica* | | | | | |
| --- | --- | --- | --- | --- | --- |
|  | Estimate | | SE | *z* | *p* |
| Intercept (Site – Namdapha) | -3.48983 | | 0.12842 | -27.175 | <0.001 |
| Site – Miao | -2.97396 | | 0.24202 | -12.288 | <0.001 |
| Contrast 1 | -0.23083 | | 0.06412 | -3.6 | <0.001 |
| Contrast 2 | 0.095 | | 0.08771 | 1.083 | 0.279 |
| Contrast 3 | 0.46144 | | 0.15313 | 3.014 | 0.003 |
|  |  | |  |  |  |
| *Dysoxylum* sp. | | | | | |
|  | Estimate | | SE | *z* | *p* |
| Intercept (Site – Namdapha) | -6.3151 | | 0.2561 | -24.663 | <0.001 |
| Site – Miao | -3.8683 | | 0.6561 | -5.896 | <0.001 |
| Contrast 1 | 0.2925 | | 0.1289 | 2.27 | 0.023 |
| Contrast 2 | 0.6628 | | 0.1902 | 3.485 | <0.001 |
| Contrast 3 | 1.3231 | | 0.3805 | 3.477 | <0.001 |
|  | | | | | |
| *Phoebe* sp. | | | | | |
|  | | Estimate | SE | *z* | *p* |
| Intercept (Site – Namdapha) | | -5.7734 | 0.3246 | -17.78 | <0.001 |
| Site – Miao | | -1.0051 | 0.5699 | -1.764 | 0.078 |
| Contrast 1 | | 0.3797 | 0.1849 | 2.054 | 0.04 |
| Contrast 2 | | 0.7015 | 0.2633 | 2.664 | 0.008 |
| Contrast 3 | | 0.5625 | 0.4683 | 1.201 | 0.22968 |
| Site: contrast 1 | | -0.8497 | 0.3379 | -2.515 | 0.012 |
| Site: contrast 2 | | -1.06 | 0.4594 | -2.307 | 0.021 |
| Site: contrast 3 | | -0.4986 | 0.7942 | -0.628 | 0.53 |
